# Supplementary material for: Shear Stress Initiates Endothelial-to-Mesenchymal Transition in Endocardial Endothelial Cells
Source: Ann Biomed Eng. 2026 Jan 28;54(5):1306–18. doi: 10.1007/s10439-026-03973-6 (PMC12866971; doi:10.1007/s10439-026-03973-6)
Supplement: Supplementary file 1 — Supplementary file1 (PDF 550 KB) [file 10439_2026_3973_MOESM1_ESM.pdf]

SUPPLEMENTARY MATERIALS

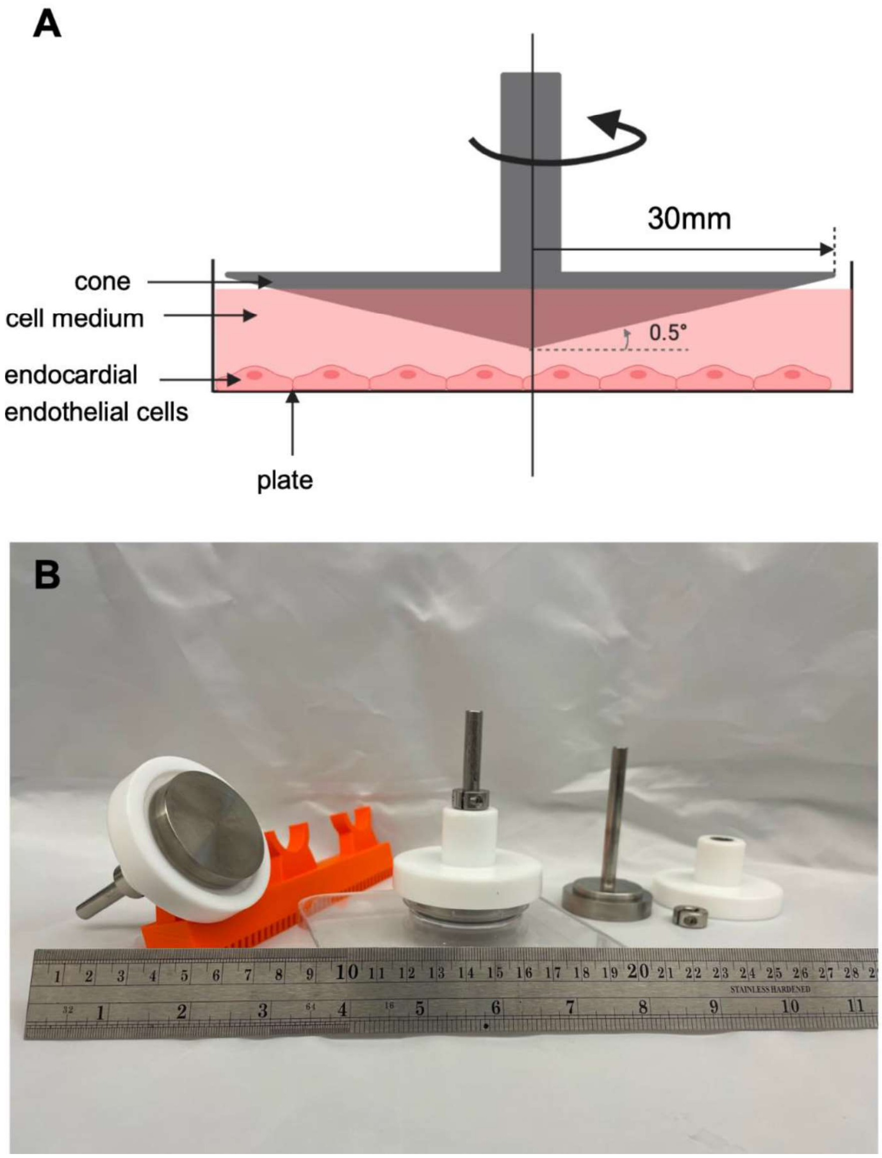

Supplemental Figure 1. Cone-and-plate Device. **(A)** Illustration of the cone-and-plate device. Endocardial endothelial cells are grown in a monolayer in a 60 mm petri dish, and the cone is loaded onto the petri dish containing the cells and cell culture medium and rotated using a controllable magnetic stir plate. **(B)** Photo of the cone and Teflon cover setup used in the shear stress studies. On the left of the photo, the cone is supported by a 3D printed holding device, which is used to position multiple cones towards a UV light for sterilization prior to use. In the center of the photo, the cone is loaded onto a petri dish. On the right of the photo, the cone, Teflon cover, and shaft collar are disassembled to show the individual parts used in the setup.

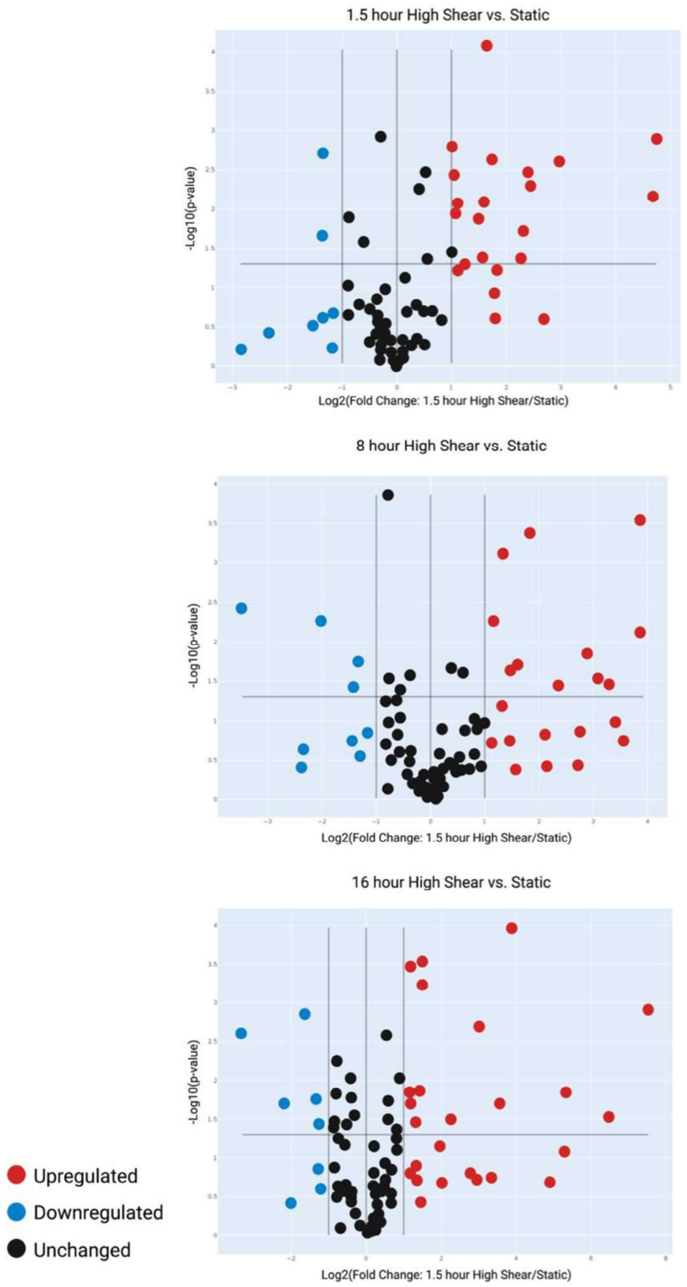

Supplemental Figure 2. Volcano plots comparing the gene expression of static EECs to EECs exposed to high shear stress at 1.5, 8, and 16 hours, respectively. Genes that are upregulated are shown as red dots, genes that are downregulated are shown as blue dots, and genes that remain unchanged are shown as black dots.

Supplemental Table 1. Cone-and-plate device settings.

**Rotational Speed Setting** (RPM)	**Shear Stress Applied** ($dynes/cm^2$)
280	15
370	20
660	35

Supplemental Table 2. Experimental conditions for shear stress experiments.

	**Static Control**	**Low Shear Stress** (15 $dynes/cm^2$)	**Medium Shear Stress** (20 $dynes/cm^2$)	**High Shear Stress** (35 $dynes/cm^2$)
**1.5 hours**	Static 1.5 hours	Low shear 1.5 hours	Medium shear 1.5 hours	High shear 1.5 hours
**8 hours**	Static 8 hours	Low shear 8 hours	Medium shear 8 hours	High shear 8 hours
**16 hours**	Static 16 hours	Low shear 16 hours	Medium shear 16 hours	High shear 16 hours

Supplemental Table 3. Taqman or Qiagen porcine primers specific to the genes of interest for PCR.

Gene of Interest	Primer Probe ID (Taqman) or GeneGlobe ID (Qiagen)
CD31	Ss03392600_u1 (Taqman)
ACTA2	Ss04245588_m1 (Taqman)
VCAM1	Ss03390912_m1 (Taqman)
ICAM1	Ss03392385_m1 (Taqman)
GAPDH	Ss03374854_g1 (Taqman)
SNAI1	PPS07880A (Qiagen)
GAPDH	PPS00192A (Qiagen)

Supplemental Table 4. Antibody information and concentrations used for flow cytometry.

Antibody Information	Amount of Antibody Added to 100 μL Sample
**Extracellular Proteins**	
CD31 Monoclonal Antibody (LCI-4) [APC] – Thermo Fisher, #MA5-16769	10 μL
Mouse IgG1 kappa Isotype Control [APC] – Thermo Fisher, #17-4714-42	5 μL
VCAM-1 Monoclonal Antibody (1.G11B1) [FITC] – Thermo Fisher, #MA5-16430	4 μL
Igg1, K Mouse, Clone: P3.6.2.8.1, Isotype Control [FITC] – Thermo Fisher, #11-4714-42	4 μL
CD62E/CD62P Monoclonal Antibody (1.2B6) [PE] – Thermo Fisher, #MA5-28649	4 μL
Mouse IgG1 Isotype Control [PE] – Thermo Fisher, #MA518097	4 μL
**Intracellular Proteins**	
Snail Antibody [AlexaFluor® 750] – Novus Biologicals, # NBP2-27293AF750	4 μL
Rabbit IgG Isotype Control [AlexaFluor 750] – Novus Biologicals, # NBP2-24891AF750	4 μL
Vimentin Antibody (RV203) [PerCP] – Novus Biologicals, # NBP1-97671PCP	4 μL
Mouse IgG1 Isotype control [PerCP] – Novus Biologicals, # IC002C	4 μL
α-Smooth Muscle Actin Antibody [AlexaFluor 405] – Novus Biologicals, # NBP2-34522AF405	4 μL
Mouse IgG2a Isotype Control (M2AK) [AlexaFluor 405] – Novus Biologicals, # NBP1-96981AF405	4 μL

Supplemental Table 5. Full data set of EEC gene expression and fold regulation in response to high shear stress (35 dynes/cm^2) at 1.5 hours, 8 hours, and 16 hours, respresented as a heatmap in Figure 4. In the Gene Symbol columns, the * symbol indicates that the gene's average threshold cycle is relatively high (>30) in either the control or the test sample, and is reasonably low in the other sample (<30), suggesting that the actual fold regulation value is at least as large as the reported fold regulation value. In the Fold Regulation columns, bolded numbers indicate that this gene exceeded the fold regulation threshold of 2, meaning this gene was upregulated (if positive value) or downregulated (negative value) in response to shear stress at this time point. In the p-value columns, bolded p-values indicate the gene expression observed is statistically significant (p<0.05) in comparison to the static control group. Genes are listed in order of the profiler array plate position. Note that genes for which the average threshold cycle was determined to be high (> 30) have been excluded from this table to ensure accuracy of reported results.

1.5 Hours			8 Hours			16 Hours		
**Gene Symbol**	**Fold Regulation**	**p-value**	**Gene Symbol**	**Fold Regulation**	**p-value**	**Gene Symbol**	**Fold Regulation**	**p-value**
ABL1	1.21	0.540121	ABL1	1.15	0.695620	ABL1*	-1.47	0.231918
ACTA2	1.31	**0.005508**	ACTA2	-1.53	0.152235	ACTA2	**2.81**	**0.000603**
AKT1	1.11	0.075017	AKT1	-1.08	0.499801	AKT1	1.14	0.071425
BCL2	-1.02	0.802490	BCL2	-1.27	0.588542	BCL2*	-1.37	0.271104
CAV1	-1.17	0.104922	CAV1	-1.12	0.777320	CAV1	-1.87	**0.014893**
CCL2	**5.38**	**0.004813**	CCL11	**-11.11**	**0.003605**	CCL11	**-4.33**	**0.019946**
CTGF	**-2.59**	**0.001949**	CCL2	-1.78	0.192672	CCL2	-1.92	0.135904
CEBPB*	**-7.25**	0.607630	CTGF	**-2.50**	**0.017989**	CTGF	1.16	0.270771
JUN	**7.95**	**0.002424**	CEBPB	-1.30	0.242663	CEBPB	1.51	0.290460
COL1A2	1.08	0.649424	JUN	1.00	0.935931	JUN	-1.50	0.254432
COL3A1	1.47	**0.046892**	COL1A2	1.36	0.429572	COL1A2	**38.86**	**0.014502**
CXCR4	-1.40	0.505999	CXCR4	-1.33	0.485514	COL3A1*	**183.81**	**0.001261**
EDN1	-1.23	0.604289	EDN1	-1.58	0.055340	CXCR4	**-2.25**	0.250346
EGF	**4.96**	**0.020138**	ENG	1.45	0.319121	DCN	**90.73**	**0.031496**
ENG	1.10	0.609845	IL1A	**8.30**	**0.030976**	EDN1	-1.73	**0.005902**
FASLG	**3.32**	**0.002393**	ILK	1.03	0.557597	ENG	1.83	**0.046697**
IL1B	**-2.57**	**0.021839**	ITGA1*	**-4.06**	**0.005391**	ILK	1.13	0.226817
ILK	-1.04	0.674167	ITGA2	1.12	0.542976	ITGA1*	**-9.85**	**0.002572**
INHBE	**4.76**	**0.048119**	LOC100517053	**3.53**	**0.000420**	ITGA2*	**-3.11**	**0.001401**
ITGA1	-1.14	0.470250	ITGAV	-1.50	**0.044684**	LOC100517053	1.55	0.154126
ITGA2	**2.05**	**0.010713**	ITGB1	1.56	0.130197	ITGAV	1.03	0.787618
LOC100517053	-1.01	0.926509	ITGB3*	**14.81**	**0.000275**	ITGB1	1.80	**0.047030**
ITGAV	-1.01	0.872866	ITGB5	-1.01	0.962301	ITGB3*	**8.02**	**0.002018**
ITGB1	1.02	0.791480	ITGB8	-1.68	0.105258	ITGB5	1.37	0.239532

1.5 Hours			8 Hours			16 Hours		
**Gene Symbol**	**Fold Regulation**	**p-value**	**Gene Symbol**	**Fold Regulation**	**p-value**	**Gene Symbol**	**Fold Regulation**	**p-value**
ITGB5	-1.19	0.301955	LOC1000 38019	-1.72	**0.028793**	ITGB8	-1.32	0.328220
ITGB8	-1.91	**0.012929**	SNAI1*	**7.60**	**0.014473**	LOC10003 8019	-1.69	0.050603
LOC10003 8019	-1.16	0.424999	LOX	-1.31	0.327202	LOX	1.45	0.126836
SNAI1*	**26.60**	**0.001352**	LOC1006 27243*	**6.86**	0.145521	LOC10062 7243*	**15.14**	**0.000107**
LOX	-1.04	0.678399	MMP14	**2.78**	**0.022457**	MMP14	**2.07**	**0.014138**
MMP14	1.09	0.488623	MMP2	-1.49	0.259701	MMP2	-1.65	0.240076
MMP2	-1.22	0.268772	MYC	-1.07	0.692770	MYC	**-2.31**	**0.035862**
MYC	**2.95**	**0.046039**	NFKB1	-1.10	0.671565	NFKB1	1.01	0.991373
NFKB1	1.27	0.173665	PDGFA	1.11	0.283864	PDGFA	-1.32	**0.009820**
PDGFA	1.44	**0.003379**	PLAT	**2.48**	**0.000870**	PLAT	1.41	0.198602
PLAT	**3.04**	**0.008234**	PLAU*	**5.05**	**0.032809**	PLAU	1.52	**0.030341**
PLAU	**2.80**	**0.013067**	SERPINE 1	1.75	0.097237	SERPINE1	**2.60**	**0.013912**
SERPINE1	**2.07**	**0.008814**	SERPINH 1	**2.48**	0.061137	SERPINH 1	1.52	**0.019464**
SERPINH1	1.01	0.831593	SMAD2	1.31	**0.021648**	SMAD2	1.44	**0.002571**
SMAD2	-1.24	**0.001113**	SMAD3	-1.02	0.848107	SMAD3	-1.97	**0.040385**
SMAD3	**2.11**	0.055446	SMAD4	1.07	0.611947	SMAD4	1.17	0.155932
SMAD4	-1.29	0.140945	SMAD6	-1.62	0.294007	SMAD6	-1.71	0.301776
SMAD6	**2.33**	**0.049408**	SMAD7	**9.70**	**0.032216**	SMAD7	**2.46**	**0.034339**
SMAD7	**25.54**	**0.006950**	SP1	1.21	0.413718	SP1	1.09	0.639097
SP1	-1.26	0.292278	STAT1	-1.72	0.056513	STAT1	-1.48	0.063858
STAT1	-1.19	0.360376	TGFB1	**2.93**	**0.020816**	TGFB1	**2.76**	**0.000298**
TGFB1	**2.01**	**0.001592**	TGFB2	1.42	0.405871	TGFB2	**4.71**	**0.032484**
TGFB2	-1.03	0.791053	TGIF1	**2.18**	**0.005368**	TGIF1	**2.14**	**0.000343**
TGIF1	**3.09**	**0.000093**	THBS1	1.07	0.661091	THBS1	**2.22**	**0.018209**
THBS1	-1.27	0.231704	TIMP1	**15.17**	**0.007376**	TIMP1	**11.32**	**0.020287**
TIMP1	**2.02**	**0.003785**	TIMP2	**-2.74**	**0.041479**	TIMP2	-1.94	**0.034846**
TIMP2	-1.38	0.190410	TIMP3	-1.75	0.713563	TIMP3	1.24	0.383076
TIMP3	-1.53	**0.028974**	VEGFA	1.49	**0.024391**	VEGFA	**2.51**	0.187627
TNF	**5.18**	**0.003202**	ACTG1	-1.48	0.090930	ACTG1	**-2.48**	**0.017117**
VEGFA	1.99	**0.034918**	B2M	-1.32	**0.028227**	B2M	-1.40	**0.036057**
ACTG1	-1.22	0.283476	GAPDH	1.15	0.129160	GAPDH	1.97	**0.009484**
B2M	1.02	0.777534	HPRT1	1.05	0.475802	HPRT1	-1.31	**0.016557**
GAPDH	1.12	0.203054	RPL13A	-1.75	**0.000138**	RPL13A	-1.29	**0.030081**
HPRT1	-1.07	0.467381						
RPL13A	-1.22	0.346220						
